# RBM20, a Therapeutic Target to Alleviate Myocardial Stiffness *via* Titin Isoforms Switching in HFpEF

**DOI:** 10.3389/fcvm.2022.928244

**Published:** 2022-06-16

**Authors:** Na Li, Weijian Hang, Hongyang Shu, Ning Zhou

**Affiliations:** Hubei Key Laboratory of Genetics and Molecular Mechanisms of Cardiological Disorders, Division of Cardiology, Department of Internal Medicine, Tongji Hospital, Tongji Medical College, Huazhong University of Science and Technology, Wuhan, China

**Keywords:** RNA binding motif protein 20, titin, myocardial stiffness, heart failure with preserved ejection fraction, splicing factors

## Abstract

Increased myocardial stiffness is critically involved in heart diseases with impaired cardiac compliance, especially heart failure with preserved ejection fraction (HFpEF). Myocardial stiffness mainly derives from cardiomyocyte- and extracellular matrix (ECM)-derived passive stiffness. Titin, a major component of sarcomeres, participates in myocardial passive stiffness and stress-sensitive signaling. The ratio of two titin isoforms, N2BA to N2B, was validated to influence diastolic dysfunction *via* several pathways. RNA binding motif protein 20 (RBM20) is a well-studied splicing factor of titin, functional deficiency of RBM20 in mice profile improved cardiac compliance and function, which indicated that RBM20 functions as a potential therapeutic target for mitigating myocardial stiffness by modulating titin isoforms. This minor review summarized how RBM20 and other splicing factors modify the titin isoforms ratio, therefore providing a promising target for improving the myocardial compliance of HFpEF.

## Introduction

Heart failure (HF) with preserved ejection fraction (HFpEF) is prevalent in an aging society and effective therapy is still unavailable for this public health issue. Augmented cardiac stiffness is an unfavorable state characterized by diastolic dysfunction and impaired cardiac compliance that is mainly involved in HFpEF (1). Increased cardiovascular stiffness plays a substantial role in the development of multiple diseases, including hypertension and heart failure (2). Echocardiography (3) and cardiac magnetic resonance imaging (CMRI) (4) have been applied to evaluate the compliance of the heart in clinical work. However, little attention has been attracted to cardiomyocyte or myofibril passive stiffness although atomic force microscopy (AFM) has been applied in laboratory experiments (5). Cardiac stiffness is dependent on cardiomyocyte-dependent and independent factors. Cardiomyocyte-dependent factors mainly derive from the elasticity of titin protein, while cardiomyocyte-independent factors are composed of cardiac fibroblasts and extracellular matrix (ECM). Both increased titin-based and collagen-dependent stiffness contribute to myocardial stiffness and excavate HFpEF (6, 7).

The largest monomer protein among identified proteins (8), titin spans across the sarcomere of striated muscle, providing mechanical support as well as passive tension to the sarcomere during the systole (9, 10). Genotypic or phenotypic mutations of titin are involved in cardiac remodeling and cardiomyopathy (11–13). However, the underlying mechanisms remain largely unclear.

Both alternative splicing and posttranslational modifications (PTMs) of titin play a pivotal role in its function (9). Apart from PTMs of titin that has been well summarized in previous reviews (14, 15), titin’s pre-mRNA is mainly modulated by RNA binding motif 20 (RBM20) (16). RBM20 is one of the splicing factors that regulate titin isoforms switching (17, 18). Two dominant titin isoforms, N2BA and NAB, differ in extensibility due to the composition of the structural domains (15). The elasticity of N2BA is higher than N2B owing to more proportion of proline-glutamate-valine-lysine (PEVK) and Ig motifs, which are the extensible elements in I-band titin (15). Myocardial compliance is modulated by different ratios of N2BA and N2B. In other words, improving the ratio of N2BA and N2B contributes to improving myocardial compliance. Previous studies showed a promising role in regulating RBM20 expression or interfering with its structure to alleviate myocardial stiffness *via* interfering with the ratio of N2BA and N2B (19, 20).

In this review, we summarized and analyzed the role of RBM20 and its co-operators in alternative splicing to regulate titin isoforms ratio, revealing a potential therapeutic target of attenuating myocardial stiffness presented in HFpEF.

## Myocardial Stiffness

Cardiac diastolic performance mainly depends on ventricular compliance, which presents a negative relationship with myocardial stiffness and accompanies by increased viscoelastic forces that resist diastolic filling (21). Increased cardiovascular stiffness impairs myocardial and vascular elasticity, and leads to insufficient organic perfusion and clinical symptoms (22). Echocardiography (3, 23) and CMRI have been used to evaluate cardiac stiffness (4). The E’, E/A ratio, left ventricle end-diastolic diameter (LVED; d), LV end-diastolic pressure (LVEDP) and pressure-volume loop evaluated by echocardiography were regarded as markers to reflect the cardiac compliance (24). LV pressure-volume (P-V) relationship, an indirect marker for ventricle compliance, was determined by LV volume and aortic pressure acquired in CMRI (4). Despite these tests, currently there is still no effective treatment for cardiac stiffness to improve cardiac compliance and flexibility. Basic research is in an urgent need to find out potential targets for improving cardiac stiffness.

The mechanism of cardiac stiffness has not been fully understood. Various pathological changes are involved in myocardial stiffness, including cardiac fibrosis (25), increased collagen in ECM (26), and most importantly, increased intrinsic cardiomyocyte stiffness which results from impairment of the cytoskeleton. Increased ECM, especially fibrillar collagen, contributes to the development of cardiac fibrosis (7, 27), which limits the systolic and diastolic function of the heart and ultimately leads to a decline in cardiac function (7). However, treatments for cardiac fibrosis have limited contribution to improving cardiac stiffness. More importantly, multiple studies over decades have revealed the substantial role of myocyte-derived compliance, which is closely related to titin (10, 28, 29). Apart from structural alterations, metabolic disorders were involved in myocardial stiffness. For instance, serum circulating proteins such as secreted frizzled-related protein 1 (sFRP1) promoted pathological changes in gene expression and cellular stiffness (30). Furthermore, coronary microvascular dysfunction, increased inflammation, oxidative stress and myocardial sodium glucose cotransporter-2 (SGLT-2)-mediated effects were significantly involved in myocardial stiffness induced by diabetic cardiomyopathy (31). Such pathological changes may lead to a decrease in energetic efficiency and suggest possible value to developing potential drugs targeting these listed mechanisms.

## The Relationship Between Titin and Cardiac Stiffness

**FIGURE 1:**
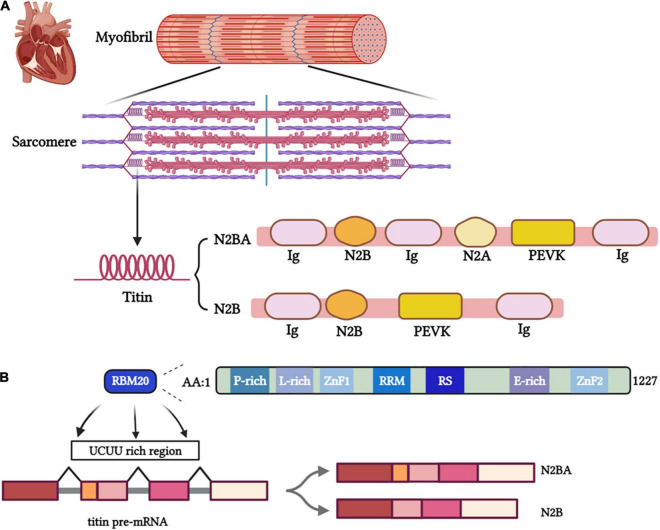
**(A)** Titin isoforms serve essential functional roles as part of the sarcomere, myofibril, and cardiac tissue. **(B)** The binding site of RBM20 on pre-mRNA and the structural and functional domains of RBM20.

The giant filament protein titin plays an essential role in facilitating the contraction of the myocardium. Titin is the largest protein in the human body encoded by 364 exons of the *TTN* gene (8, 32). Composed of four main structural domains, titin crosses sarcomeres to connect the Z-disk and the M-line and Serves as a physiological spring (Figure 1A) (29). The main extensible region of titin is composed of three domains: N2Bus, the spring-like PEVK domain and the immunoglobulin (Ig)-like domains. The PEVK and N2B regions hold a spring-like function (33), while a shortened Ig domain resulted in titin-based cardiac diastolic dysfunction (34), besides, Unfolding and refolding of the Ig domain exert different impacts on myocardial elasticity (35–37).

Studies demonstrated that Ig-like domains affect titin elasticity through redox modification (38) and S-glutathionylated (35) in the unfolded state. In detail, S-glutathionylation of cryptic cysteines enhances titin elasticity by blocking protein folding in human cardiomyocytes (35).

Titin provides structural support and elastic forces to heart tissue while being stretched. Length and viscoelasticity are predominant components of the passive tension during the heart systaltic process and the major contributors to the Frank-Starling mechanism (28, 29). The alternative splicing process produce different isoforms of titin, including adult N2BA, adult N2B and fetal cardiac titin (FCT) that are composed of different proportion of domains (Ig, PEVK, N2Bus and N2A), which differ in length and elasticity. The long, soft N2BA isoform (3,200–3,500 kDa) and the short, rigid N2B isoform (3,000 kDa) are expressed in adult human hearts (Figure 1A). Distensibility of different isoforms mainly depends on the length of spring N2B and PEVK regions (39). The longer PEVK and N2Bus expressed in N2BA isoform determined its softer property than N2A. The N2BA: N2B ratio has a pronounced impact on myocardial stiffness (40). Different titin isoforms ratio was shown to affect cardiac phenotypes and heart diseases (13, 41). The titin-isoform shift may be beneficial for myocardial diastolic function but could impair the contractile performance in systole (42). A larger proportion of N2BA is expressed in the dilated cardiomyopathy (DCM) patients’ hearts than the normal ones, accompanying by impaired cardiac systolic function (42). However, some studies demonstrated that upregulating compliant titin isoforms in a murine model with HFpEF-like symptoms improved diastolic function resulting in greater tolerance to exercise (13). Further studies need to make the mechanism clear and explore the potential therapeutics modulating the titin isoform ratio to alleviate myocardial stiffness.

PTMs are well-studied to modulate titin stiffness *via* phosphorylation and dephosphorylation (10, 15). Elasticity of titin was regulated by activating PKG or PKCα at the N2B element with its extensible unique sequence (N2Bus) or PEVK region (9, 43). Myocardial stiffness was, respectively, decreased and increased by phosphorylating the N2Bus and PEVK region of titin (44, 45). Phosphoserines within N2Bus, including P-S4010, P-S4099 and P-S4062, are independently activated by ERK1/2 (46), cAMP-dependent PKA (47), cGMP-dependent PKG and CaMKII (14, 47, 48). In contrast, phosphorylating the PEVK region of titin by PKC exacerbated myocardial stiffness (49). Accordingly, regulating the phosphorylation of N2Bus and PEVK domains may attenuate cardiac stiffness induced by chronic heart diseases (CHD).

## Splicing Factors Play an Essential Role in Modulating Titin Isoforms

### RBM20, a Silver Lining for Patients With Cardiac Diastolic Dysfunction

Mis-splicing of titin mitigated by RBM20 was markedly involved in heart diseases (50, 51). As a striated muscle-specific gene located on chromosome 10, the RBM20 gene contains 14 exons and encodes a protein of 1,227 amino acids mostly expressed in the myocardium (52, 53). RBM20 contains two zinc finger domains, an RNA recognition motif (RRM), a serine- and arginine-rich region (RS region), a leucine-rich region, and a glutamate-rich region (Figure 1B) (54). Two pivotal functional regions, RRM and RS region play a dominant role in the nuclear localization of RBM20. Meanwhile, the most frequent pathogenic RBM20 mutations occur in the RS region.[55] RBM20 binds with introns near splice sites and adjacent to U1 and U2 small nuclear ribonucleoprotein (snRNP) binding sites to regulate splicing (53). Haploinsufficiency of RBM20 led to altered alternative splicing of *TTN* and a dramatic shift to highly compliant titin isoforms and impaired Frank-Starling mechanism (55). Moreover, the serine and arginine residues in the RS region (RSRSP stretch) can be phosphorylated, which is necessary for the nuclear localization of RBM20. Loss of phosphorylation of these mutated residues promoted the translocation of RBM20 from the nucleus and negated the function of RBM20 (56). Given a better understanding of regions of RBM20 (57), it is intriguingly to elucidate how mutation of RBM20 affect cardiac function *via* regulating splicing of pre-mRNA of titin. RBM20 represses splicing to orchestrate cardiac pre-mRNA processing. In failing human hearts, reduced expression of RBM20 affects alternative splicing of several direct targets, indicating that differences in RBM20 expression may affect cardiac function (53).

RBM20 plays an essential role in titin isoform switching (16). Genotype mutations or functional site alterations in RBM20 lead to different outcomes (57, 58). For instance, genetic mutations (59) and mutations in RS domain (60) of RBM20 were observed to closely associated with familial DCM cases. In addition, a mutation in the glutamate-rich region of RBM20 causes DCM through missplicing of titin and impaired Frank–Starling mechanism (55). More descriptions of the relationship between RBM20 variation and corresponding exons and pathogenicity were summarized in previous reviews (61). Subendocardial fibrosis accompanied by electrical abnormalities has been observed in RBM20-null heterozygous or homozygous rats (58). RBM20 deficiency in rats leads to many phenotypic features that are observed in individuals with cardiomyopathy related to mutant RBM20, suggesting conserved RBM20 function. Researchers found that RBM20 was a global regulator of cardiac alternative splicing and document considerable overlap of post-transcriptionally regulated genes that depend on RBM20. They offer mechanistic insights and functional annotation of RBM20 substrates that contribute to cardiomyopathy and heart failure (58).

Among the cardiac genes regulated by RBM20, *TTN* is a major human disease-causing one (50, 58). DCM was closely related to mutations in RBM20 (59). A previous study demonstrated that RBM20-mutated (a missense mutation in the RSRSP stretch) mice caused the development of DCM (62). However, researchers experimentally promoted the compliance of titin by RBM20 inhibition with a mouse model termed cRbm20ΔRRM, referred to as one of the RRM of the RBM20 alleles was floxed and which expressed the MerCreMer transgene under control of the αMHC promoter (63). Inhibiting the RBM20-based titin splicing system contributed to upregulating compliant titin and improving diastolic function in an HFpEF model (63). In addition, increased ventricular stiffness and diastolic dysfunction in N2B-KO mice were reversed by a 50% reduction of functional RBM20 expression (64), which relied on mechanical properties of titin and broadened the therapeutics of cardiac stiffness mediated by RBM20 (64).

The latest study revealed that inhibition of RBM20 with antisense oligonucleotides (ASOs) in HFpEF mouse and engineered human heart tissue improved diastolic function (20), which indicated a marked translational value of RBM20 in attenuating myocardial wall stiffness. Myocardial stiffness was estimated by pressure-volume catheter and or AFM and also reflected by increased radial and circumferential strains (65). Myocardial compliance was reflected by the velocity of LV pressure varying with LV volume in the P-V curve, while AFM assesses the elastic and adhesive behavior of cardiomyocytes through topography detection to the exploiting advanced nanomechanical mapping (66). AFM increasingly provides a thorough insight into cardiac physiological and pathological conditions, as well as the effects of therapeutic approaches at the cardiomyocytes scale.

RBM20 is crucial for the formation of a subset of circRNAs that originate from the I-band of the titin gene (67). CircRNAs affect splicing (68) and regulate gene expression (69). Khan et al. identified 80 different circRNAs within the *TTN* gene and determined RBM20-sensitive exons served as a substrate for circRNA formation when they were spliced out of the linear titin transcript (67).

### RBM20 Coordinates With RBM24 to Regulate Cardiac Hypertrophy

RNA Binding Motif 24 (RBM24), a member of the RNA binding protein (RBP) family, is also an important splicing factor involved in titin splicing. The RBM24 full and conditional knockout mice were embryonic lethal and exhibited DCM or heart failure, respectively (70). RBM24 deletion in mouse model resulted in misconnection of genes encoding structural proteins of sarcomere, such as *Tpm2*, *TTN*, *Neb1*, *Fhod3*, *Enah*, and *Ablim1* (71). As a major regulator of muscle-specific alternative splicing, RBM24 is necessary for sarcomere assembly and cardiac contraction (72). Latest study showed that RBM24 modulates the temporal dynamics of core myofibrillogenesis genes and thereby orchestrates sarcomere organization *via* facilitating inclusion of exon 6 of *ACTN2* (73). In addition, RBM24 promoted the inclusion of exons 11 and 13 of *TTN*, which located on the Z-disk and participate in the assembly, stabilization, and maintenance of myofibrils (70, 74). More research are called for indicating mechanisms involved in RBM24 regulating *TTN* and myocardial fibrils assembly in the future.

Intriguingly, RBM20 and RBM24 were shown to co-regulate the splicing of scaffold proteins expressed by the *ENH* genes in rat cardiomyocytes (75). In healthy conditions, RBM20 and RBM24 cooperate to promote the expression of short *ENH* isoforms (*ENH3*), which prevents hypertrophic growth. However, RBM20 or RBM24 alone had no significant effect on the alteration of scaffold protein subtypes. In addition, promoting RBM20 and RBM24 increased the expression of *ENH* subtypes lacking LIM domains (such as *ENH3* and *ENH4*), thus prevented myocardial hypertrophy in mice (75). It is reasonable to speculate that the cooperatively regulating RBM20 and RBM24 may affect myocardial stiffness, because cardiac hypertrophy is closely related to wall stiffness and compliance, but the mechanisms remain unknown.

Therefore, future studies are needed to determine whether exist potential mechanism that RBM20 and RBM24 cooperate to regulate myocardial stiffness by muscle-specific alternative splicing.

### The Mechanism of the Regulation of the Titin Isoforms Conversion by RBM20 and PTB4

RBM20 splicing upon the post-transcriptional precursor mRNA of the *TTN* gene effectively regulated cardiomyocyte stiffness (28). The specific mechanism may be related to the phosphorylation of the RS domain, the deletion of the glutamate-rich domain, and the change in plasma hormone levels. Determining the *TTN* pre-mRNA binding sites is the key to regulating the proportion of titin subtypes by RBM20.

As a splicing inhibitor near exons, RBM20 Predominantly binds to intron sequences containing UCUU motifs (Figure 1B) (52, 58). Recent studies have confirmed RBM20 uses a coupled folding binding mechanism by the C-terminal helix to specifically recognize the UCUU RNA motif (76).

The novel titin splicing factor PTB4 (alias PTBP1) is a member of the heterogeneous nuclear ribonucleoprotein (hnRNP) family that expressed in the heart, skeletal muscle, and brain (77). PTB4 binds to UC-rich regions in introns on both sides of regulatory exons (78). PTB4 and RBM20 jointly regulated the splicing of cardiac precursor mRNA (17). Interestingly, PTB4 acts as a novel regulator of titin splicing in exon exclusion through counteracting the RBM20 repressor activity (79). PTB4/RNA complexes are formed only with RNAs containing UCUU motifs. Dauksaite et al. showed that PTB4 and RBM20 bind to the transcriptional sequence with the same motif on the 5′SS downstream of the alternative exon (79). This provided a possible mechanism of regulating titin isoform expression through additive binding of PTB4 and RBM20 to the downstream intron (79). It is of great significance to figure out RBM20 and PTBP4 as the potential therapeutic targets for attenuating myocardial stiffness and diastolic dysfunction.

As one of the main inhibitors of splicing events, PTB4 inhibited specific exons in alternative splicing isomers (80). The expression of RBM20 and PTB4 increased and decreased during cardiac development, respectively. This contrary trend of PTB4 and RBM20 during heart development showed an inhibition effect of PTBP1 on the splicing effect of RBM20 on titin (17, 81).

In addition, both PTB4 and RBM20 are involved in regulating the alternative splicing of formin homologous 2 domains containing 3 (FHOD3) proteins, the sarcomere proteins regulating the dynamics of actin, thus participating in actin assembly and sarcomeric organization of cardiomyocyte (81). Further study of alternative splicing of FHOD3 by RBM20 and PTB4 may contribute to exploring mechanisms and therapeutic targets of cardiomyopathy.

## Titin Isoforms Switching Is Modulated by Hormone Stimuli *Via* Modulating RBM20 Expression

**FIGURE 2:**
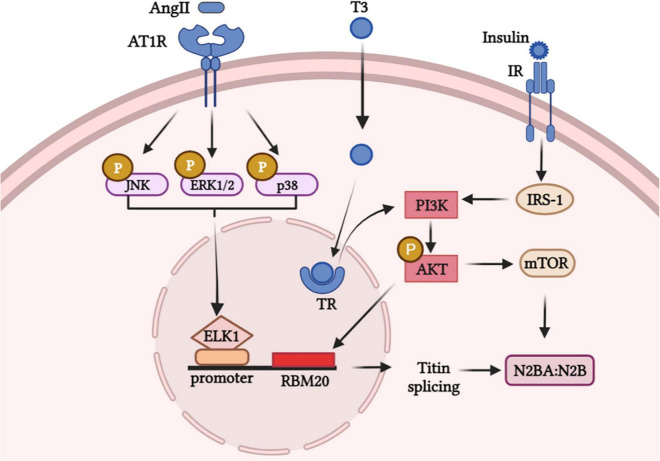
Hormone stimuli promote signaling pathways in modulating the expression of titin isoforms *via* RBM20.

RBM20 was demonstrated to be involved in the process of multiple external stimuli regulating titin isoforms transition, thus modulating the myocardial wall stiffness. Here, we summarized signaling pathways that linking factors and RBM20 to regulate titin isoforms switching (Figure 2). For instance, titin isoform transition triggered by the thyroid hormone-triiodothyronine (T3) is linked to RBM20 *via* the PI3K/Akt/mTOR signaling pathway, phosphorylating RBM20 and/or increasing gene expression of RBM20 (18). No N2B isoform was expressed in RBM20^–/–^ rat hearts under T3 treatment relative to control groups implanted with placebo, while propylthiouracil (PTU) treatment attenuated N2BA:N2B ratio in RBM20^+/+^ rats (18). Furthermore, researchers revealed that mechanism linking titin isoforms and T3 mainly due to the PTMs (specifically phosphorylation) of RBM20 (18).

Besides, insulin controls titin-based cardiac stiffness *via* activating PI3K/Akt/mTOR pathway and increasing titin phosphorylation (82). Researchers showed the mean proportion of the stiffer N2B-titin isoform significantly increased in insulin-treated primary neonatal rat cardiomyocytes (NRCMs) and diastolic function was improved in diabetic cardiomyopathy (82). Likewise, the central position of RBM20 in PI3K/Akt/mTOR pathway for insulin-induced titin alternative splicing was validated in another study (83). However, whether Akt directly act on the SR rich region or phosphorylate RBM20 to regulate splicing of titin remains unclear and worth further exploration.

AngII has been demonstrated to activate MAPK-ELK axis and to upregulate the expression of RBM20 (84). This study revealed that Ang II can trigger ELK1 through activation of MAPK signaling by enhancing RBM20 expression which regulates pre-mRNA splicing. After AngII binds to AngII receptor 1 (AT1R), intracellular molecules including JNK/ERK1/p38 are phosphorylated and the downstream transcription factor ELK1 translocated into the nucleus. ELK1 was activated and combined with titin pre-mRNA, which triggered the expression of RBM20 and regulated the titin isoforms ratio (84).

Furthermore, RBM20 methylation was decreased and the RBM20 mRNA level increased upon doxorubicin treatment (85). Accordingly, further studies need to reveal signaling pathways involved in RBM20 and unravel the potential therapeutic targets of augmented cardiac stiffness.

Better understanding the mechanisms involved in titin isoforms transition shows a silver lining for treating heart failure with myocardial wall stiffness.

## Conclusion

HFpEF characterized by diastolic dysfunction remains a serious public health problem with high morbidity and mortality. Novel therapeutics or prevention strategies are urgent for solving this problem. Titin, the spring protein of sarcomere in myocardium, plays an essential role in controlling compliance and elasticity during contractile relaxation. Modification of titin isoforms ratio *via* splicing factor RBM20 represents a potential target to attenuate myocardial stiffness. As effective regulators of cardiac stiffness based on titin, RBM20 provides novel strategies to treat heart diseases with impaired cardiac compliance, especially HFpEF. RBM24 and PTB4 broadened the splicing scope of titin, which provided new therapeutics and is worthy of further exploration.

Both RBM20 heterozygous mouse model (86) and reducing RBM20 activity in N2B-KO mouse (64) inhibited titin-based stiffness and diastolic dysfunction and improved cardiac function. However, patients that carry a pathogenic RBM20 mutation have more ventricular arrhythmias comparing to patients with a *TTN* mutation (87). Haploinsufficiency of RBM20 disturbed alternative splicing of *TTN* and resulted in a dramatic shift to highly compliant titin isoforms and an impaired Frank-Starling Mechanism (55). Titin isoform analysis revealed a dramatic shift from the less compliant N2B toward the highly compliant N2BA isoforms in RBM20*^E913K/^*^+^ heart that carrying a mutation in a glutamate-rich region of RBM20. These effects may contribute to the early onset, and malignant course of DCM caused by RBM20 mutations (55). As those side effects exist, more research are urgently needed to translate the clinical therapy value of RBM20.

Besides, multiple small molecules modulate titin isoforms switching, *via* RBM20-dependent or independent way. C-type natriuretic peptide (CNP) modulated titin-based ventricular compliance (88) and Digoxin attenuated RBM20 protein level (89), respectively. Cardenolides affect RBM20-dependent titin isoforms expression *via* decreasing RBM20 protein levels and altering transcription of select splicing factors that interact with RBM20 (90). It is promising that taking small molecules to inhibit the splicing activity of RBM20 (90).

In summary, our mini review has illustrated a promising way to attenuate myocardial stiffness characterized in heart failure, which mainly depends on the role of splicing factor RBM20 on titin pre-mRNA to modulate titin isoforms ratio.

## Author Contributions

NL wrote the original draft. NZ, WH, and HS revised the manuscript. All authors agreed to be accountable for the content of the work.

## Conflict of Interest

The authors declare that the research was conducted in the absence of any commercial or financial relationships that could be construed as a potential conflict of interest.

## Publisher’s Note

All claims expressed in this article are solely those of the authors and do not necessarily represent those of their affiliated organizations, or those of the publisher, the editors and the reviewers. Any product that may be evaluated in this article, or claim that may be made by its manufacturer, is not guaranteed or endorsed by the publisher.
